# Supplementary material for: High level of soluble tumor necrosis factor receptors is associated with lower residual diuresis volume in patients on hemodialysis: An exploratory study
Source: PLoS One. 2025 Apr 29;20(4):e0320019. doi: 10.1371/journal.pone.0320019 (PMC12040094; doi:10.1371/journal.pone.0320019)
Supplement: S1 Dataset — The dataset includes the following variables: Height, Lean Mass (kg), LMI (kg/m²), Fat Mass (kg), FMI (kg/m²), Duration of HD (years), Diuresis Volume (mL), Kt/V, Body Fat (%), A/G Ratio, Visceral Fat (g), BMD Total (g/cm³), BMD Spine (g/cm³), BMD Hip (g/cm³), Leptin (ng/mL), sTNF-R1 (pg/mL), and sTNF-R2 (pg/mL). (PDF) [file pone.0320019.s001.pdf]

Participant	Heigth	Lean Mass (kg)	LMI (kg/m2)	Fat mass (kg)	FMI (kg/m2)	Duration of HD (years)	Diuresis Volume *(mL)*	Kt/v	Body fat (%)	a/g ratio	Visceral fat (g)	BMD total (g/cm3)	BMD spine (g/cm3)	BMD hip *(g/cm³)*	Leptin *(ng/mL)*	sTNF-R1 *(pg/mL)*	sTNF-R2 *(pg/mL)*
1	1.53	26.48	11.31	16.29	16.29	2.67	50	1.78	38	0.72	300	0.92	0.85	0.73	1708.4	2915.56	1848.11
2	1.54	32.52	13.71	11.67	4.92	5.00	0	1.63	26	0.48	424	0.88	0.85	0.7	166.26	3521.08	2337.92
3	1.54	38.70	16.32	28.74	12.12	3.83	800	1.37	43	1.03	2174	0.89	0.94	0.66	1691.95	2448.01	1995.42
4	1.83	71.70	21.41	35.60	10.63	2.2	425	1.0	33	1.66	690	1.11	1.06	0.88	1556.98	3879.8	2708.95
5	1.61	45.82	17.68	36.64	14.14	0.92	150	1.2	44	1.07	1091	1.10	1.38	0.88	1600.73	3665.18	2507.32
6	1.73	55.62	18.58	12.56	4.20	1.67	0	1.21	18	1.18	1333	1.05	0.89	0.82	993.79	1830.22	1918.09
7	1.47	42.51	19.67	10.38	4.80	17.3	0	1.72	24	0.69	1005	0.76	1.16	0.59	90.75	2723.94	2230.2
8	1.64	52.07	19.36	20.37	7.57	1.17	250	1.23	28	1.62	318	1.39	1.51	1.21	1315.29	2218.06	2139.05
9	1.60	35.81	13.99	10.39	4.06	5.25	1550	1.69	23	0.57	412	0.93	0.97	0.82	201.51	1477.64	1966.88
10	1.69	46.37	16.24	6.88	2.41	6.5	100	1.4	13	0.48	579	0.93	0.91	0.78	438.26	2921.69	1916.25
11	1.79	51.96	16.22	11.01	3.44	2.75	200	1.15	18	0.6	284	1.17	1.15	0.98	136.63	2914.03	2166.67
12	1.50	41.29	18.35	40.82	18.14	1.58	350	1.33	38	1.17	694	1.01	1.12	0.91	69.91	2073.96	1756.05
13	1.44	28.67	13.83	11.06	5.33	5.75	0	1.6	28	0.66	1660	0.72	0.64	0.49	350.93	2205.8	1834.3
14	1.57	39.43	16.00	15.05	6.11	9.67	50	1.56	28	1.15	382	0.79	0.83	0.64	496.75	2949.29	1899.67
15	1.70	50.90	17.61	15.30	5.29	0.25	2000	0.71	23	0.95	3144	1.06	1.02	0.84	195.46	2601.3	2514.69
16	1.68	42.51	15.06	10.57	3.75	8.5	0	1.46	20	0.68	650	1.12	1.22	0.95	314.53	3902.79	2192.45
17	1.48	28.59	13.05	12.66	5.78	1.25	850	1.21	31	0.67	2107	0.85	0.97	0.73	1667.91	1286.02	1879.42
18	1.68	44.35	15.71	17.92	6.35	1.5	100	1.17	29	1.03	2282	1.08	1.03	0.93	1215.52	2872.64	1872.05
19	1.66	48.37	17.55	16.50	5.99	2	3250	1.16	25	1.51	756	0.96	0.99	0.85	942.74	1974.32	1642.8
20	1.66	50.56	18.35	22.90	8.31	8.5	0	1.34	31	1.7	215	1.16	1.34	1.08	1455.28	3038.2	2124.32
21	1.52	33.31	14.42	14.06	6.09	9.7	0	1.5	30	0.57	1135	0.88	0.89	0.72	748.19	3594.67	2398.68
22	1.57	40.27	16.34	6.91	2.80	6.08	0	1.63	15	0.6	9	0.76	0.83	0.63	69.67	3283.47	2513.77
23	1.52	29.16	12.62	11.44	4.95	4.58	100	1.67	28	0.71	2453	0.74	0.71	0.47	851.58	2670.29	1830.62
24	1.72	66.04	22.32	12.27	4.15	0.42	300	1.13	16	0.68	1119	0.80	0.92	0.58	171.83	2418.88	1993.58
25	1.66	48.03	17.43	11.20	4.06	2.75	1650	1.3	19	0.79	455	0.95	0.87	0.81	223.01	2633.5	2153.78
26	1.72	42.93	14.51	17.36	5.87	6.25	0	1.33	29	1.84	1693	1.17	1.4	0.98	1689.63	3479.69	2358.17
27	1.65	41.67	15.31	25.13	9.23	2.17	20	1.46	38	1.13	1484	1.04	1.09	0.82	116.3	3508.82	2151.94
28	1.67	47.08	16.88	10.15	3.64	9.75	0	1.24	18	0.67	481	0.75	0.77	0.65	165.06	4594.16	2614.12
29	1.71	47.00	16.07	16.83	5.76	0.58	0	1.21	26	0.95	423	1.20	1.07	1.06	1192.49	2440.34	2161.15
30	1.70	45.71	15.82	27.01	9.35	4.33	700	1.35	37	1.47	2185	1.19	1.22	0.96	1251.61	1802.63	1507.46
31	1.76	45.49	14.69	5.54	1.79	15.5	0	1.62	11	0.67	124	1.00	1.49	1.06	90.37	5277.86	2656.47
32	1.58	39.43	15.79	39.42	15.79	1.75	2100	1.36	45	1.47	3.555	1.02	1.23	0.8	1406.31	1074.47	1471.56
33	1.77	60.81	19.41	16.05	5.12	0.92	0	1.11	21	0.86	1206	1.30	1.16	1.06	391.94	3904.32	2894.93
34	1.62	45.65	17.39	11.22	4.28	2.08	200	1.22	20	1.02	832	1.02	1.06	0.79	505.11	2264.05	2249.53
35	1.72	55.02	18.60	7.29	2.46	1.83	300	1.29	12	0.56	59	1.17	1.13	0.96	0	2881.84	1898.75
36	1.68	47.35	16.78	6.97	2.47	7.17	0	1.28	13	0.62	103	1.00	0.99	0.82	84.98	1767.37	2033.17
37	1.44	35.60	17.17	13.60	6.56	1.80	1250	1.74	27	0.99	335	0.91	0.89	0.79	54.83	1581.88	1747.76
38	1.59	47.47	18.78	11.03	4.36	4.70	0	1.35	19	0.62	424	0.86	0.83	0.66	259.99	1981.99	1609.66
39	1.70	54.18	18.75	23.98	8.30	1.50	2200	1	31	1.54	2174	1.27	1.37	1.23	1107.55	1722.92	1807.6
40	1.53	32.14	13.73	19.30	8.24	4.08	50	1.81	38	0.87	690	0.65	0.72	0.45	606.9	3660.58	2529.42
41	1.56	48.35	19.87	37.14	15.26	1.92	450	1.54	48	0.94	1091	1.03	1.04	0.95	1955.37	3355.52	2509.16
42	1.55	40.90	17.02	32.59	13.57	4.17	0	1.51	44	0.92	1433	1.27	1.42	1.19	1446.36	3337.13	2151.94
43	1.59	38.75	15.33	23.91	9.46	1.50	2400	1.24	38	1.16	1005	1.02	0.96	0.86	1664.53	1966.66	1928.21
44	1.56	31.84	13.08	31.84	13.08	2.42	200	0.98	35	0.88	318	0.91	0.91	0.77	1733.73	2963.08	2144.57
45	1.55	34.55	14.38	19.75	8.22	2.42	800	0.85	36	0.74	412	0.89	0.93	0.65	1341.59	1480.71	2122.48
46	1.52	34.91	15.11	15.99	6.92	5.16	0	1.44	31	0.72	579	0.68	0.77	0.51	732.34	4387.21	2297.41
47	1.52	30.47	13.19	10.13	4.38	14.3	0	1.54	25	0.6	284	0.79	0.84	0.7	431.51	3979.44	2667.52
48	1.61	49.30	19.02	17.06	6.58	4.25	450	3.31	26	1.18	794	1.15	1.29	0.95	1506.7	2823.04	2120.02
49	1.60	34.31	13.40	19.77	7.72	2.08	950	1.41	37	1.29	1780	1.11	1.16	0.78	1176.19	1350.41	1222.97
50	1.53	30.60	13.07	8.20	3.50	19.25	200	1.83	21	0.81	382	0.93	0.8	0.71	591.26	2615.1	2186
51	1.47	38.39	17.77	40.91	18.93	5.42	1400	1.58	52	1.28	3344	0.93	0.99	0.85	1533.44	3646.79	2604.91
52	1.54	39.92	16.83	30.60	12.90	2	100	1.19	43	1.01	650	1.30	1.39	1.14	1014.33	3396.91	2446.56
53	1.75	59.38	19.39	24.34	7.95	8.92	0	1.07	29	1.38	2171	1.15	1.29	1.07	364.37	3544.08	2476.02
54	1.54	43.17	18.20	22.12	9.33	1.5	500	1.01	34	1.61	2382	1.12	1.3	0.92	1753.63	3337.13	2381.19
55	1.77	48.37	15.44	15.31	4.89	2	0	1.29	24	0.88	756	1.02	0.96	0.82	1330.36	3286.54	2143.65
56	1.66	39.94	14.49	8.54	3.10	2.17	550	1.59	18	0.6	215	0.91	0.73	0.63	1035.23	2821.08	2129.1
57	1.42	25.81	12.80	28.48	14.12	1.4	50	2.25	53	1.13	1135	0.88	0.96	0.8	1783.21	4014.7	2597.55
58	1.43	31.52	15.41	5.82	2.85	14.08	0	1.65	16	0.42	9	1.07	1.13	1.07	311.13	1784.23	1790.11
59	1.64	45.48	16.91	22.55	8.38	9	0	1.35	33	1.72	2.653	1.05	1.08	0.92	1546.12	3200.69	2038.7
60	1.40	33.41	17.05	20.39	10.40	1.75	350	1.67	38	1.17	1119	0.77	0.86	0.6	1387.67	2012.65	1760.65
