# Supplementary material for: High level of soluble tumor necrosis factor receptors is associated with lower residual diuresis volume in patients on hemodialysis: An exploratory study
Source: PLoS One. 2025 Apr 29;20(4):e0320019. doi: 10.1371/journal.pone.0320019 (PMC12040094; doi:10.1371/journal.pone.0320019)
Supplement: Attachment — Submitted filename: Response to Reviewers.pdf [file pone.0320019.s004.pdf]

MINISTÉRIO DA EDUCAÇÃO
UNIVERSIDADE FEDERAL DOS VALES DO JEQUITINHONHA E MUCURI
DIAMANTINA – MINAS GERAIS - BRASIL

Brazil, 2025.

Title: High level of soluble tumor necrosis factor receptors is associated with lower residual diuresis volume in patients on hemodialysis: An exploratory study. (PONE-D-24-26287R1).

Section Editor - Dr. Te-Ling Lu - PLOS ONE

Dear Editor,

We have considered all the referees' suggestions and modified the manuscript accordingly. The answers to the issues the referees pointed out, and a revised version of the manuscript are enclosed (**all new text modifications are letters highlighted in yellow**).

We trust that the revised version of our manuscript will be acceptable for publication.

The authors of this manuscript declare that they have not submitted this paper for consideration by any other journal. They also affirm that no conflicts of interest are associated with this paper and that all authors have read and approved the manuscript.

Ana Cristina Rodrigues Lacerda, PhD.
Centro Integrado de Pós-Graduação e Pesquisa em Saúde (CIPq-Saúde)
Universidade Federal dos Vales do Jequitinhonha and Mucuri (UFVJM)
Campus JK – Rodovia MGT, 367 – Km 583 – N°. 5000 – Alto da Jacuba
CEP 39100 000, Diamantina, Minas Gerais – Brazil.
Tel.: + 55/38/35321283 Email: lacerdaacr@gmail.com

Comments to the Author

Reviewer: 2

Comments:

The authors addressed the comments satisfactorily with one exception which regards the following comment:

Comment 5: Indicate the type of vascular access used for HD in Tables 1 and 2. Depending on vascular access type distribution between the groups, include the vascular access in regression analysis and Table 4.

Author's Response: Thank you for your consideration. The patients utilized either a central catheter or an arteriovenous fistula. While the type of vascular access is known to be associated with the patient's clinical prognosis (Yeh et al., 2019), it does not directly correlate with residual kidney function.

Reviewer's Recommendations: The type of vascular access used for hemodialysis can significantly impact serum levels of inflammatory markers. Hence, the recommendation to include the vascular access in the regression analysis remains very important.

Response:

We appreciate the reviewer's comments. The review of the database sample revealed that, although it was not mentioned as an exclusion criterion in this study, none of the eligible patients were using a catheter. Additionally, a previous study conducted by our group (1) established arteriovenous fistula access as the sole inclusion criterion, reflecting a methodological approach adopted by the research group.

The manuscript also included information according to the reviewer's remark. Page 8, Lines 176-177: All eligible patients underwent hemodialysis through an arteriovenous fistula.

Reference

1 Santos, L.M.M., Figueiredo, P.H.S., Silva, A.C.R. et al. Determining factors of functioning in hemodialysis patients using the international classification of functioning,

disability and health. BMC Nephrol 23, 119 (2022). https://doi.org/10.1186/s12882-022-02719-5

<u>Reviewer: 3</u>

Comments:

The authors investigated the relationship between inflammatory markers and residual renal function in a cohort of 60 hemodialysis patients, of which 27 (45%) were anuric. Their findings revealed that elevated sTNFR1 levels were linked to reduced residual urine volumes, even after accounting for confounding factors.

The study is well-presented, and its conclusions are robust. Given the significance of these findings in translational research, this article warrants consideration for publication.

Response: Thank you for considering our study for publication.
