# Supplementary material for: High level of soluble tumor necrosis factor receptors is associated with lower residual diuresis volume in patients on hemodialysis: An exploratory study
Source: PLoS One. 2025 Apr 29;20(4):e0320019. doi: 10.1371/journal.pone.0320019 (PMC12040094; doi:10.1371/journal.pone.0320019)
Supplement: Attachment — Submitted filename: Response to Reviewers.docx [file pone.0320019.s003.docx]

|  | **MINISTÉRIO DA EDUCAÇÃO**  **UNIVERSIDADE FEDERAL DOS VALES DO JEQUITINHONHA E MUCURI**  **DIAMANTINA – MINAS GERAIS - BRASIL** |  |
| --- | --- | --- |

Brazil, 2024.

**Title:** High level of soluble tumor necrosis factor receptors is associated with lower residual diuresis volume in patients on hemodialysis: An exploratory study. **(**PONE-D-24-26287)

Section Editor - Dr. Te-Ling Lu - PLOS ONE

Dear Editor,

We have considered all the referees' suggestions and modified the manuscript accordingly. The answers to the issues the referees pointed out, and a revised version of the manuscript are enclosed (**all** **new** **text** **modifications** **are** **letters highlighted in yellow**).

We trust that the revised version of our manuscript will be acceptable for publication.

The authors of this manuscript declare that they have not submitted this paper for consideration by any other journal. They also affirm that no conflicts of interest are associated with this paper and that all authors have read and approved the manuscript.

**Ana Cristina Rodrigues Lacerda, PhD.**

Centro Integrado de Pós-Graduação e Pesquisa em Saúde (CIPq-Saúde)

Universidade Federal dos Vales do Jequitinhonha and Mucuri (UFVJM)

Campus JK – Rodovia MGT, 367 – Km 583 – N°. 5000 – Alto da Jacuba

CEP 39100 000, Diamantina, Minas Gerais – Brazil.

Tel.: + 55/38/35321283 Email: [lacerdaacr@gmail.com](mailto:lacerdaacr@gmail.com)

**Comments to the Author**

**Reviewer: 1**

**Comments:**

While I think it is an interesting analysis, the lack of longitudinal data is regrettable. I think the conclusions drawn from the work are not sufficiently corroborated by the data since it only reports a cross-sectional association between inflammatory markers and residual renal function.

**Response:** Thank you for your consideration. Although our study was a cross-sectional design, limiting the extrapolation of the data, no previous studies investigated the hypothesis that elevated systemic chronic inflammation may deteriorate residual renal function, as evidenced by lower residual diuresis volume in patients on hemodialysis, considering body composition measured with precise techniques and taking into account confounding factors such as gender, age, hemodialysis duration, Kt/V, and body composition. The text was modified to improve the conclusion.

**Modified text:** page 16, lines 333-339.

**Reviewer: 2**

**Comments:**

This study looked at the relationship between serum inflammation-related molecules and the level of residual kidney function in a cross-sectional cohort of patients on chronic HD.

**Comment 1:** Indicate why only patients who have been on HD for more than 6 months were included.

**Response:** End-stage renal disease (ESRD) is characterized by kidney damage and loss of kidney function lasting more than three months (Lameire et al., 2021; KDIGO, 2024). We included only patients who had been on hemodialysis for over six months to ensure stable levels of residual kidney function. During the initial phase of hemodialysis, patients often experience significant fluctuations due to the adaptation period. By selecting these patients, we aimed to minimize such variations and create a more homogeneous study population, thereby enhancing the reliability of our data. The same selection criteria were utilized in previous studies (Santos et al., 2022; Figueiredo et al., 2017).

**References:**

1. Lameire NH, Levin A, Kellum JA, et al. Harmonizing acute and chronic kidney disease definition and classification: report of a Kidney Disease: Improving Global Outcomes (KDIGO) Consensus Conference. Kidney Int. 2021;100:516–526. doi: 10.1016/j.kint.2021.06.028.
2. Kidney Disease: Improving Global Outcomes (KDIGO) CKD Work Group. KDIGO 2024 Clinical Practice Guideline for the Evaluation and Management of Chronic Kidney Disease. Kidney Int. 2024 Apr;105(4S):S117-S314. doi: 10.1016/j.kint.2023.10.018.
3. Santos LMM, et al.[Determining factors of functioning in hemodialysis patients using the international classification of functioning, disability and health.](https://pubmed.ncbi.nlm.nih.gov/35331148/) BMC Nephrol. 2022. Mar 24;23(1):119. doi: 10.1186/s12882-022-02719-5.
4. Figueiredo PH, et al. [The role of the inspiratory muscle weakness in functional capacity in hemodialysis patients.](https://pubmed.ncbi.nlm.nih.gov/28278163/) PLoS One. 2017. Mar 9;12(3):e0173159. doi: 10.1371/journal.pone.0173159. eCollection 2017.

**Comment 2:** Indicate why you chose to look at residual kidney function as a binary variable and why the cut-off of 100ml/day. Consider running the analyses with residual kidney function as a continuous variable.

**Response:** Residual diuresis may be defined as a urinary output greater than 100 mL per day or a glomerular filtration rate exceeding 1 mL per minute (de Sequera et al., 2017; Termorshuizen et al., 2004; Rodrigues et al., 2024). In the initial analysis, we aimed to determine whether there were differences in biomarker concentrations between anuric individuals (<100 mL) and non-anuric individuals (>100 mL). Subsequent analyses evaluated the linear relationship between diuresis volume (a continuous variable) and inflammatory biomarkers.

**References:**

1. de Sequera P, Corchete E, Bohorquez L, Albalate M, Perez-Garcia R, Alique M, et al. Residual Renal Function in Hemodialysis and Inflammation. Therapeutic apheresis and dialysis : official peer-reviewed journal of the International Society for Apheresis, the Japanese Society for Apheresis, the Japanese Society for Dialysis Therapy. 2017;21(6):592-8. Epub 2017/10/04. Doi: 10.1111/1744-9987.12576.
2. Termorshuizen F, Dekker FW, van Manen JG, Korevaar JC, Boeschoten EW, Krediet RT, et al. Relative contribution of residual renal function and different measures of adequacy to survival in patients on hemodialysis: an analysis of the Netherlands Cooperative Study on the Adequacy of Dialysis (NECOSAD)-2. J Am Soc Nephrol. 2004;15(4):1061-70. Doi:10.1097/01.asn.0000117976.29592.93.
3. Rodrigues VGB, Silva WT, da Silva ACR, Campos PC, Santos LMM, Lacerda ACR, Alves FL, Maciel EHB, Prates MCSM, Costa HS, de Lima VP, Mendonça VA, Figueiredo PHS. Relationship between residual diuresis and sarcopenia in patients on hemodialysis. J Ren Nutr. 2024 Jun 22:S1051-2276(24)00138-9. doi: 10.1053/j.jrn.2024.06.006.

**Comment 3:** What was the average interval (in days, weeks, months, or years) between the DEXA study and the testing of residual kidney function?

**Response:** According to the study protocol, researchers assessed residual kidney function the day before the first weekly hemodialysis session and conducted the DEXA scan immediately after the second weekly session. As a result, the average interval between these two assessments was three days.

**This information is available in:**

Page: 6, lines 133-134; Page:7, line 154-155.

**Comment 4:** What was the average interval (in days, weeks, months, or years) between the measurement of sTNF-R1 and sTNF-R2 and the testing of residual kidney function?

**Response:** According to the study protocol, researchers assessed the inflammatory biomarkers during the second weekly hemodialysis session and evaluated residual kidney function the day before the first weekly hemodialysis session. Therefore, the average interval between the two assessments was three days.

**This information is available in:** Page 6, lines 133-134. page 7, lines 140-141.

**Comment 5:** Indicate the type of vascular access used for HD in Tables 1 and 2. Depending on vascular access type distribution between the groups, include the vascular access in regression analysis and Table 4.

**Response:** Thank you for your consideration. The patients utilized either a central catheter or an arteriovenous fistula. While the type of vascular access is known to be associated with the patient's clinical prognosis (Yeh et al., 2019), it does not directly correlate with residual kidney function.

**Reference:**

1. Yeh LM, Chiu SY, Lai PC. The Impact of Vascular Access Types on Hemodialysis Patient Long-term Survival. Sci Rep. 2019 Jul 24;9(1):10708. doi: 10.1038/s41598-019-47065-z. PMID: 31341241; PMCID: PMC6656721.

**Comment 6:** The authors state: “During the study, patients underwent HD sessions using low-flux dialysis membranes,” Clarify what ‘during the study’ means, how long the patients were in the study.

**Response:** Thank you for your consideration. The patients participated in the study cross-sectionally, with each variable assessed only once during the protocol week. The phrase "during the study" was misleading, as the hemodialysis unit where the research was conducted strictly follows this protocol. The unit opted for low-flux membranes because they are better tolerated by patients. We have made the suggested changes.

**Modified text:** page 6, line 116.

**Comment 7:** Before entering the study, were any patients on high-flux dialyzers? If so, how many HD sessions, on average, did they receive with high-flux dialyzers before being switched to low-flux dialyzers?

**Response:** Before entering the study, no patient had undergone sessions with high-flux dialyzers. The unit adopted only the low-flux membrane because it is better tolerated by the patients.

**Comment 8:** Were other urine tests obtained, such as urea nitrogen or creatinine, and if so, please report Kidney stdKt/V or kidney urea clearance, kidney creatine clearance, in Tables 1 and 2.

**Response:** Measuring daily urine volume is the simplest method for evaluating residual renal function (Moist et al., 2000). Research has demonstrated that daily urine volume correlates with the residual glomerular filtration rate (GFR) (references below). Therefore, we did not conduct additional urine tests, as prior studies have employed the same approach.

**This information is available**: Pages 6-7, lines 133-138.

**References:**

1. Moist LM, Port FK, Orzol SM, et al. Predictors of loss of residual renal function among new dialysis patients. J Am Soc Nephrol. 2000;11:556–564. doi:10.1681/ASN.V11355613.
2. Lemes HP, Araujo S, Nascimento D, et al. Use of small doses of furosemide in chronic kidney disease patients with residual renal function undergoing hemodialysis. Clin Exp Nephrol. 2011;15:554–559. doi:10.1007/s10157-011-0427-z14.
3. Iwasawa H, Nakao T, Matsumoto H, Okada T, Nagaoka Y, Wada T. Phosphate handling by end-stage kidneys and benefits of residual renal function on phosphate removal in patients on haemodialysis: phosphate handling by RRF in HD patients. Nephrology. 2013;18:285–291. doi:10.1111/ nep.12039.
4. Dopierała M, Schwermer K, Hoppe K, Kupczyk M, Pawlaczyk K. Benefits of Preserving Residual Urine Output in Patients Undergoing Maintenance Haemodialysis. Int J Nephrol Renovasc Dis. 2023 Oct 17;16:231-240. doi: 10.2147/IJNRD.S42153

**Comment 9:** Consider adding in the Discussion that, besides the fact that this study does not prove causality, the direction of the association is also uncertain, For example, high levels of inflammatory markers can be a consequence (at least partly) of low residual kidney function rather than the etiology of low residual kidney function, or both.

**Response:** Thank you for your consideration. Changes have been made to the manuscript discussion according to the reviewer's suggestion.

**Modified of text:** page 15, lines 306-312.

**Comment 10:** Limitations appear in two separate sections –on page 15 and 16. Needs to be consolidated into one section, before Conclusions.

**Response:** Thank you for the suggestion. Changes have been made to the manuscript limitation according to the reviewer's observation.

**Modified of text:** page 16, lines 318-332.

**Comment 11:** Same for Conclusions, it needs to be consolidated, it is mentioned on page 15, ‘Inc conclusion,’ and again the paragraph on page 16.

**Response:** Thank you for the suggestion. Changes have been made to the manuscript conclusion according to the reviewer's observation.

**Modified of text:** page 16, lines 333-339

**Comment 12:** Reference 11 – did not cross over properly, missing authors, journal.

**Response:** Thank you for your consideration. Changes have been made to the manuscript references, and authors have been added correctly according to the reviewer's observation.

**Modified of text:** page 19.

**Comment 13:** Tables 3 and 4: a/g ratio: add in the legend what a/g denotes.

**Response:** Thank you for your consideration. According to the reviewer's observation, changes have been made to the manuscript tables 3-4.

**Modified of text:** page 11.
